# Cesarean Delivery of a Parturient with Lumboperitoneal Shunt under Spinal Anesthesia

**DOI:** 10.1155/2023/8892695

**Published:** 2023-07-22

**Authors:** Hicham Ziani, Med Yassir Lahbabi, Nabil Elachhab, Nawfel Elcaidi, Aziz Slaoui, Hanaa Lazhar, Sophia Lahbabi, Aziz Baidada, Nezha Oudghiri, Rajae Tachinante

**Affiliations:** ^1^Department of Intensive Care Unit, Maternity Hospital IBN SINA, Rabat, Morocco; ^2^Department of Gynecology, Maternity Hospital IBN SINA, Rabat, Morocco

## Abstract

Lumboperitoneal shunt may be indicated as a treatment for idiopathic intracranial hypertension aiming to facilitate the dynamic flow of cerebrospinal fluid into the peritoneum for patients. Parturients with lumboperitoneal shunt are a few, making it difficult to choose the analgesic or anesthetic technique for delivery. We present the case of a successful spinal anesthesia for a cesarean delivery in a parturient who was diagnosed with idiopathic intracranial hypertension that was treated by lumboperitoneal shunt.

## 1. Introduction

Idiopathic intracranial hypertension (IIH) usually occurs in women of reproductive age. The incidence is 1/100,000 for normal-weight women but increases to 20/100,000 for obese women. Intracranial pressure is elevated (>250 mm H_2_O); the exact cause is unknown but probably involves cerebral venous flow obstruction. The diagnosis is mainly made by magnetic resonance imaging and lumbar puncture.

Headache, vision problems (transient vision loss, double vision), and pulsatile tinnitus are the most common symptoms. Permanent loss of vision is the most severe consequence. Blindness is definitive even if the intracranial pressure is reduced.

The treatment is mainly medical and aims to reduce the intensity of headaches, intracranial pressure, and preservation of vision. If vision worsens despite treatment, a lumboperitoneal (LP) shunt may be indicated.

Parturients with IIH or with an LP shunt are few, making it difficult to choose the analgesic or anesthetic technique for delivery.

We report in this observation, the case of a parturient followed for IIH carrying an LP shunt and in whom an indication of cesarean delivery was established. The anesthetic technique was spinal anesthesia.

## 2. Patient and Observation

### 2.1. Patient Information

**Figure 1:**
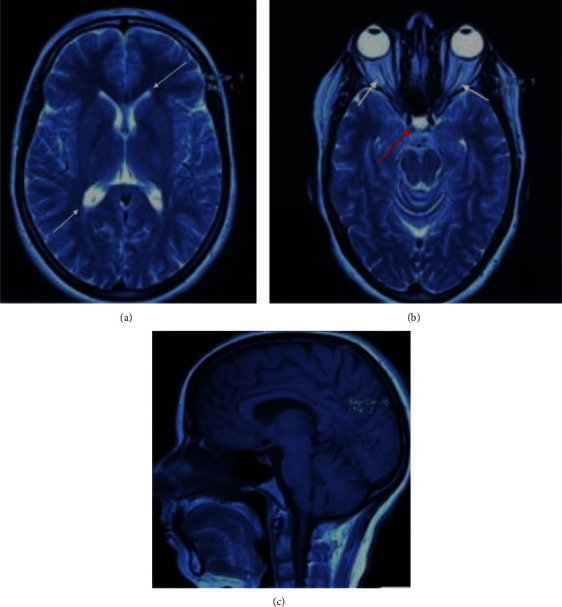
(a) T2 axial sequence MRI showing normal-sized lateral ventricles (white arrow), (b) axial T2 sequence showing optic nerve sheath enlargement (white arrow) with an empty aspect of the sella turcica (red arrow) suggesting intracranial hypertension, and (c) T1 sagittal sequence showing discrete ptosis of the cerebellar tonsils.

**Figure 2:**
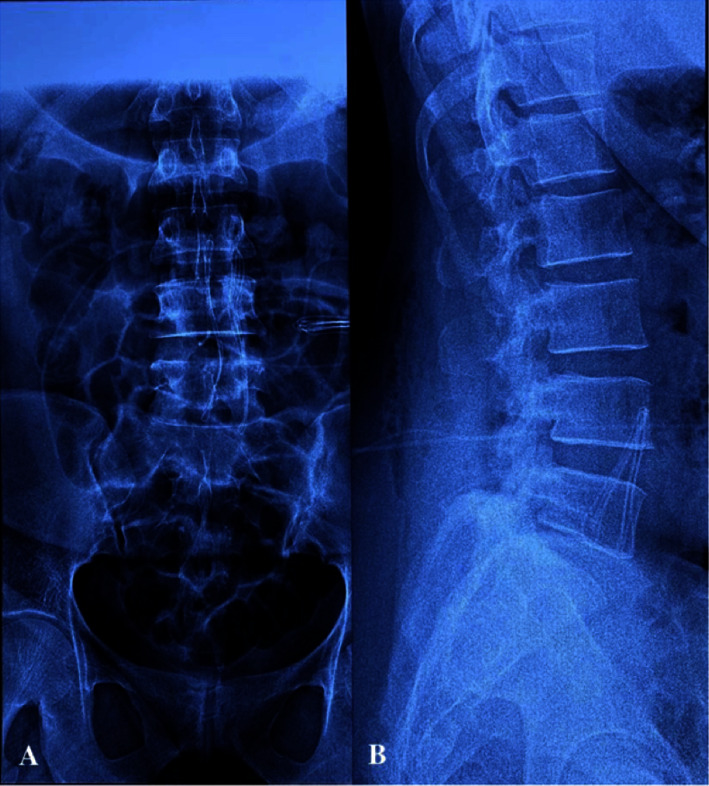
Postoperative radiographic control of the front (A) and side (B) showing the location of the tip of the catheter at the intrarachid level, introduced at the level of L4-L5, and which communicates with its distal tip positioned in the peritoneal cavity.

A 36-year-old parturient, gravida 4 para 3, 40 weeks of gestation, followed in neurosurgery for 2 years for an IIH (Figure 1) managed with LP derivation at the level of the L4-L5 space (Figure 2) after failure of the medical treatment (based on acetazolamide) and decrease of the vision.

The patient's interview revealed that she had not taken any medication during her pregnancy.

Given the medical history of the parturient of IIH, a vaginal delivery under epidural analgesia was indicated during the pregnancy follow-up.

### 2.2. Clinical Findings

The patient was admitted to the emergency room of the maternity hospital for uterine contraction and labor and moderate pain (5 points on the Visual Analogue Scale (VAS)). The obstetric examination showed acute fetal distress on the fetal heart rate tracing requiring an urgent cesarean section. Bimanual vaginal examination found that the cervix was dilated by 2 cm and effaced by 60%.

The preanesthetic examination found a conscious apyretic patient well oriented in time and space, without headache, nor vomiting in jet or diplopia, blood pressure at 12/7 cmHg, heart rate at 80 bpm, eupnea and normal blood sugar level at 1.1 g/l, body mass index at 28 kg/m^2^ (weight 74 kg, height 163 cm), with criteria for difficult intubation, Mallampati III and thyromental distance at 4.5 cm.

**Figure 3:**
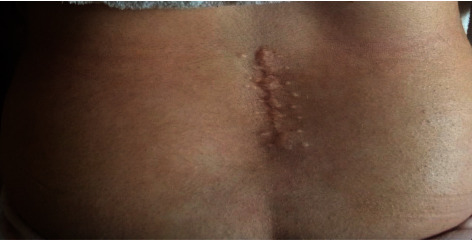
Image showing the postoperative dorsal skin scar.

Examination of the spine revealed that the spinous processes were clearly visible, and there was a cicatrix of the LP shunt between L4 and S2 (Figure 3), without any infection or inflammation adjacent to it. The location of the catheter was verified on scans from 10 months ago.

Preoperative blood tests showed hemoglobin at 13 g/dL, platelets 220000/*μ*L, leukocytes 10500/*μ*L, and prothrombin time (PT) 84%.

### 2.3. Therapeutic Interventions

The patient was admitted to the operating room and monitored in a half-seated position. Spinal anesthesia was performed at the L3-L4 level using a 22-gauge fine needle, above the skin incision scar for the LP shunt, to avoid any potential damage to the surgical shunt. The anesthesia consisted of 10 mg of hyperbaric bupivacaine 5% and 0.1 mg of morphine.

Extraction of a male newborn (Apgar 9-10-10) was performed after the complete establishment of bilateral T4 motor and sensory block.

The patient remained stable without any incident during the entire procedure, lasting approximately 90 minutes in total.

During the entire procedure, the patient maintained a stable blood pressure between 11 and 13/5-6 cmHg with a heart rate between 70 and 90 bpm, and oxygen saturation (SpO_2_) remained at 100% under 2 liters per minute of oxygen.

The motor block disappeared two hours after the spinal anesthesia.

### 2.4. Follow-Up and Outcome of Interventions

The postoperative course was simple without complication, and the daily clinical examination of the patient did not reveal any signs of intracranial hypertension: headache, vomiting, or visual problems.

The patient was discharged from the hospital 72 h later, with enoxaparin-based thromboprophylaxis and paracetamol for pain.

A neurosurgical check-up was requested after 1 month of delivery that was strictly normal with a functional LP shunt, in place, on a control scan.

## 3. Discussion

Delivery of patients with a ventriculoperitoneal cerebrospinal fluid shunt can be done through the vaginal route, in the absence of obstetrical contraindications. It is advisable in some cases not to prolong expulsive efforts, particularly in the case of a malfunction of the shunt system.

A cesarean section will only be suggested in patients with severe intracranial hypertension (ICH).

The obstetrical indication for a cesarean delivery complicates the choice of anesthetic technique in parturient with a history of benign intracranial hypertension or hydrocephalus.

It has been proven that spinal anesthesia is safe and effective for patients with ICH or benign hydrocephalus without prior LP shunt [1, 2].

However, in a patient with a pre-existing LP shunt, the anesthetic may leak into the peritoneum through the shunt leading to inadequate anesthesia [1].

In the case of pregnant patients with a pre-existing LP shunt, general anesthesia for cesarean section has been recommended over epidural anesthesia because of potential damage to the shunt during epidural placement [1]. However, two cases of successful epidural anesthesia without damage to the shunt have been reported separately [3, 4]. In addition, general anesthesia in pregnant patients, especially those with obesity, involves multiple risks, including aspiration and airway problems, and should generally be avoided if possible [4, 5].

Abouleish et al. used spinal anesthesia for a patient with an LP shunt [1].

Since the lumbar puncture was to be performed to measure cerebrospinal fluid (CSF) pressure and obtain a CSF sample for examination, the choice was to use spinal anesthesia. They noted that the spread of a local anesthetic in a patient with high CSF pressure is not known. The dermatomal level reached for this patient was not different from a patient at full term with normal CSF pressure [6]. In addition, the regression of anesthesia in this case was normal [7]. Therefore, the increase in CSF pressure may not be an important factor in determining the diffusion of a local anesthetic or the duration of spinal anesthesia.

Palop et al. [8] also found that the spread of local anesthetic after epidural analgesia in two patients with IIH was similar to the one in normal patients.

Epidural anesthesia for cesarean section in patients with LP shunt requires special considerations. Firstly, to minimize potential shunt damage, the space chosen for epidural catheter placement should be picked away from the level of the LP shunt. Since the intrathecal placed LP shunt tubing is usually tunneled subcutaneously in a lateral direction, the medial approach to the epidural technique should be used. Although radiological exploration to localize the LP shunt before epidural placement can be useful [5], it should not be mandatory.

Tarshis et al. [9] also considered the risk to be minimal when, without prior imaging, they inserted an epidural needle under the scar of a parturient with an implanted intrathecal pump.

Kaul et al. [10] described the case of accidental spinal anesthesia in a patient with an LP shunt during the placement of an epidural catheter for vaginal delivery, and it was believed that a leakage of local anesthetics into the peritoneal cavity occurred via the LP shunt because of the rapid offset of local anesthetic action requiring additional lidocaine reinjections.

In our case, the spinal needle was inserted above the scar and the location of the catheter after radiological verification, without leakage of the local anesthetic given the quality of the sensitive and motor blocks, and without damage of the shunt after radiological confirmation by the neurosurgeon and the absence of clinical signs suggesting a recurrence of intracranial hypertension.

## 4. Conclusion

The choice of anesthetic technique in parturients with an LP shunt must be discussed in a collegial manner and based on clinical and radiological criteria.

Spinal anesthesia remains an alternative to general anesthesia, especially in the case of obese parturients with difficult intubation criteria requiring an urgent delivery via C-section in the absence of clinical or radiological signs of intracranial hypertension.

## Data Availability

The radiographic data used to support the findings of this study are included within the article as well as the patient's photos. All data used to support the findings of this study are available from the corresponding author upon request.

## Additional Points

*The Following Is Already Known.* (i) The main risks of locoregional anesthesia in a patient with a lumboperitoneal shunt: peritoneal leakage of local anesthetics, insufficient anesthesia, or higher than desirable motor block level. (ii) Spinal anesthesia in a patient with a lumboperitoneal shunt has been rarely described in the literature. *This Study Provides the Following*. (i) The interest in presenting this case is based on the successful use of spinal anesthesia in a parturient with a lumboperitoneal shunt during an upper abdominal delivery. (ii) By *presenting* this case, we discuss this anesthetic technique as an alternative to general anesthesia, especially in patients with difficult ventilation or intubation criteria.
